# Tumor Angiogenesis in the Absence of Fibronectin or Its Cognate Integrin Receptors

**DOI:** 10.1371/journal.pone.0120872

**Published:** 2015-03-25

**Authors:** Patrick A. Murphy, Shahinoor Begum, Richard O. Hynes

**Affiliations:** Howard Hughes Medical Institute, David H. Koch Institute for Integrative Cancer Research, Massachusetts Institute of Technology, Cambridge, MA, United States of America; UT-Southwestern Med Ctr, UNITED STATES

## Abstract

Binding of α5β1 and αvβ3/β5 integrin receptors on the endothelium to their fibronectin substrate in the extracellular matrix has been targeted as a possible means of blocking tumor angiogenesis and tumor growth. However, clinical trials of blocking antibodies and peptides have been disappointing despite promising preclinical results, leading to questions about the mechanism of the inhibitors and the reasons for their failure. Here, using tissue-specific and inducible genetics to delete the α5 and αv receptors in the endothelium or their fibronectin substrate, either in the endothelium or globally, we show that both are dispensable for tumor growth, in transplanted tumors as well as spontaneous and angiogenesis-dependent RIP-Tag-driven pancreatic adenocarcinomas. In the nearly complete absence of fibronectin, no differences in vascular density or the deposition of basement membrane laminins, ColIV, Nid1, Nid2, or the TGFβ binding matrix proteins, fibrillin-1 and -2, could be observed. Our results reveal that fibronectin and the endothelial fibronectin receptor subunits, α5 and αv, are dispensable for tumor angiogenesis, suggesting that the inhibition of angiogenesis induced by antibodies or small molecules may occur through a dominant negative effect, rather than a simple functional block.

## Introduction

Extracellular matrix proteins and their adhesion receptors are enticing targets for the regulation of tumor angiogenesis. The recruitment of new blood vessels by tumors is an important bottleneck in tumor development, without which tumors fail to grow. Thus, targeting tumor angiogenesis has been a therapeutic goal. Endothelial cell migration and survival is strongly regulated *in vitro* by adhesion to extracellular matrix, mediated by integrin receptors on the endothelium. Since the endothelium and its underlying matrix are readily targeted with small molecules and antibodies, disrupting matrix-integrin interactions would seem to be a useful method of inhibiting tumor angiogenesis.

Interactions between the extracellular matrix protein Fibronectin (FN) and its integrin receptors were some of the first such proposed targets, since FN and its receptors are strongly expressed around the tumor vasculature, and both are essential for developmental angiogenesis. Embryos and embryoid bodies deficient in FN fail to form vascular networks, despite proper endothelial cell specification and vasculogenesis of the dorsal aorta and cardinal vein [1–3]. The FN binding integrins include α5β1, α4β1, α8β1, α9β1, αvβ1, αvβ3, αvβ5, αvβ6 and αvβ8 [4]. Embryos deficient in the α5 subunit (Itga5) of α5β1, considered the primary FN receptor, are embryonic lethal with vascular defects [5]. Combined deletion of integrin αv (Itgav) and α5 results in a more severe phenotype than deletion of αv alone, yielding a spectrum of defects resembling the FN-null embryos and suggesting that these two alpha subunits contribute to the primary FN receptors in embryonic vascular development [6]. Indeed, mutating the RGD motif in FN critical for binding of both α5β1 and αvβ/3β5 integrin receptors also results in embryonic lethality with vascular phenotypes [7]. Thus, several lines of genetic evidence suggest that binding of FN by α5- and αv-based integrins is critical for mammalian angiogenesis.

One of the critical processes regulated by the FN-binding integrins is the assembly of soluble FN into insoluble FN fibrils [8]. *In vitro* experiments suggest this is an essential step in incorporation of other matrix proteins, such as the fibrillins, latent-TGFβ-binding proteins, collagens, and elastin, and the subsequent development of the endothelial basement membrane [8]. Blocking FN assembly also disrupts vascular network formation *in vitro* and in collagen plugs *in vivo*, suggesting that establishment of the proper, FN-based basement membrane is essential for angiogenesis [9]. Although FN assembly increases in co-cultured endothelial and mural cells, which cell type is responsible for the assembly remains unclear [10]. Both are independently capable of FN production and assembly *in vitro*, and the same may be true *in vivo*. The complete endothelial deletion of α5 and αv did not significantly interfere with developmental angiogenesis or FN assembly *in vivo*, although isolated endothelial cells exhibited major defects in FN assembly *in vitro*, and most of these mice eventually succumbed to embryonic defects in the remodeling of the great vessels [11]. Likewise, deletion of α5 in smooth muscle cells and pericytes did not obviously affect vascular development, although it did cause defects in lymphatic valve remodeling [12]. Thus, FN assembly appears to be essential for the formation of the basement membrane and angiogenesis, but the cells type(s) critical for *in vivo* assembly during angiogenesis remain unclear.

Although early preclinical studies supported the utility of inhibitors of the FN- α5β1 and FN- αvβ3/β5 interactions, the clinical results thus far have been disappointing. The most advanced study to date, a Phase III clinical trial of the selective αvβ3 and αvβ5 integrin inhibitor Cilengitide revealed no treatment benefit [13]. A competitive inhibitor of the α5β1 synergy site important in FN binding, ATN-161, also moved to Phase II clinical trials, but there are no ongoing studies with this drug [14]. Antibodies targeting α5β1 more specifically have been no more successful. Volociximab, designed to bind α5β1 and block interactions with FN, did not result in significant therapeutic benefits in several clinical trials—some of which were discontinued for failing to reach primary thresholds [14]. PF-04605412, also designed to bind α5β1, failed to reach primary thresholds, despite effective suppression of tumor growth when used in preclinical xenografts [15]. It is difficult to know whether such treatments would have worked if the inhibition obtained were complete. In fact, low doses of Cilengitide have been shown to promote, rather than suppress, tumor angiogenesis [16]. Higher and more consistent doses are possible in pre-clinical models, suggesting the possibility that the level of inhibition achieved, rather than the target, may be the reason behind the potent pre-clinical effects and disappointing clinical results. Genetic mutation of the genes involved would help to resolve these questions, but due to the embryonic lethality of the α5 knockout, studies to date have involved teratocarcinomas (which recruit host vasculature) [17] and heterozygous or mosaic deletion of α5, neither of which fully addresses the requirement for this integrin in the vasculature of tumors [18]. Likewise, while portions of the FN protein [19] or plasma pool [20] have been genetically removed, the effect of complete FN ablation on tumor angiogenesis and matrix deposition has not been examined.

Here, to determine the absolute requirement for the FN-integrin interactions in tumor angiogenesis, we used genetic tools to remove the FN-binding integrin subunits α5 and αv from the endothelium, prior to tumor growth, in two transplant models and the RIP-Tag model of angiogenesis-dependent pancreatic cancer. We further tested the requirement for FN, from any source, in RIP-Tag tumors in mice with global post-natal deletion of FN.

## Materials and Methods

### Mice

Integrin *Itga5* ^*f/f*^ *; Itgav* ^*f/f*^ (also called *α5* ^*f/f*^ *; αv* ^*f/f*^) mice were generated from an intercross of our existing *Itga5* ^*f/f*^ and *Itgav* ^*f/f*^ lines, as we previously reported [11, 21]. *FN* ^*f/f*^ mice were obtained from R. Fassler [22]. To obtain endothelial deletion of integrins α5 and αv, or FN, the respective lines were crossed with *Cdh5-CreER* ^*T2*^ (obtained from R. Adams [23]) or *ROSA-CreER* ^*T2*^ (obtained from T. Jacks [24]) mice. Cre-mediated excision efficiency was monitored with the fluorescent *mT/mG* Cre-reporter mice [25], crossed with the *FN* ^*f/f*^ mice. The reporter switches from red to green when activated by Cre. *RIP1-Tag2* mice were obtained from the National Cancer Institute [26], and intercrossed with the *α5* ^*f/f*^ *; αv* ^*f/f*^, *FN* ^*f/f*^, *Cdh5-CreER* ^*T2*^, *ROSA-CreER* ^*T2*^, and *mT/mG* mice.

In experiments with *Cdh5-CreER* ^*T2*^ mice, excision was induced with 3 x 1mg Tamoxifen by intraperitoneal injection one week before tumor transplant, or at weeks 5–6 in RIP-Tag mice. Excision in *ROSA-CreER* ^*T2*^ mice was induced with 5 x 1mg Tamoxifen by intraperitoneal injection at weeks 5–6 in RIP-Tag mice, and then either continued with 1 x 1mg weekly, or not.

All mice were housed and handled in accordance with approved Massachusetts Institute of Technology Division of Comparative Medicine protocols (IACUC approval 0412-033-15).

In brief, animals are monitored daily by animal care staff and laboratory personnel and appropriate measures are taken if infection, inflammation or distress occur. If body condition score is <2 or if aggregate tumor burden exceeds 1 cm in diameter, mice are euthanized by CO2 narcosis. We use a regulated flow valve (in addition to cylinder regulator) that restricts the CO2 flow rate to 20% of chamber volume per minute. These methods were chosen because they cause minimal stress to the animal. All methods of euthanasia are consistent with the recommendations of the Panel on Euthanasia of the American Veterinary Medical Association.

### Tumor transplant and quantitation

Lewis lung carcinoma (LL2, ATCC) or melanoma (B16/F10, ATCC) cells were injected subcutaneously on each dorsal flank, delivering either 1x10^5^ or 5x10^5^ cells to littermate mutant and control mice. Tumors were harvested 12 days (LL/2) or 18 days later (B16/F10) and weighed.

### RIP-Tag tumor quantitation

Blood-filled angiogenic tumors were dissected from the pancreas of RIP-Tag mice at 10–11 weeks or 12–13 weeks of age and counted (10–11 weeks) or weighed (12–13 weeks).

### RNA isolation and quantitation

RNA was extracted using the Qiagen RNAeasy mini-column kit, after combining the chloroform extract from Trizol 1:1 with 70% ethanol. A Promega kit was used to random prime a cDNA library from each tumor before expression analysis using the Bio-Rad iQ sybr mix (Table 1).

**Table 1:** Primers for quantitative PCR.

Gene	Forward	Reverse
Murine 18s	GTAACCCGTTGAACCCCATT	CCATCCAATCGGTAGTAGCG
Murine FN	CTTTGTGGTCTCATGGGTCTC	AGCAGGTCAGGAATGTTCAC
Murine αv	TGGAACCTGCTTTCTTCAGG	CTACCAGGACCACCGAGAAG
Murine α5	TTCTCCGTGGAGTTTTACCG	GGTGGTGCACTGGATAGGAC
Genomic Murine Control	AACAAGAAAGGCCTCACTTCAG	GGTGTTCTCTGACTTATACATGCAC
Genomic Murine FN *No amplification product upon Cre-mediated excision*	GTGTGAGCCGGACAACTTC	TCACGCTTGCTCTGACTGAC

### Staining and fluorescence quantification

For quantification of fluorescence, tumors were fixed in 4% PFA, sucrose infused and snap frozen in Optimal cutting temperature compound (OCT, Tissue Tek) on a metal block cooled by liquid nitrogen, and then sectioned at 10μm thickness. Sections blocked in 2% BSA, 0.1% TritonX-100 in PBS were stained using the primary antibodies indicated (Table 2). All samples were stained and imaged in parallel for experiments in which comparisons are made.

**Table 2:** Antibodies used for immunofluorescence staining.

Antibody	Source	Dilution
Anti-FN	Rabbit polyclonal, Richard Hynes Lab	1:100
Anti-α5	BD (553319, clone 5H10-27)	1:200
Anti-ColIV	Abcam (ab6586)	1:100
Anti-Nid-1	Abcam (ab14511)	1:200
Anti-Nid-2	Abcam (ab14513)	1:200
Anti-laminin	Abcam (ab11575)	1:200
Anti-Fibrillin-1	Rabbit polyclonal, Lynn Sakai Lab (9543)	1:200
Anti-Fibrillin-2	Rabbit polyclonal, Lynn Sakai Lab (868)	1:200
Andi-CD31	BD (550274, clone MEC 13.3)	1:100

Quantitation of staining area was performed in ImageJ. An arbitrary threshold was set, and the percentage of area above this threshold was quantified.

### Statistics

Student's t test was used to compare means of two independent groups to each other.

## Results

### Tumor growth is not affected by the absence of endothelial integrin subunits Alpha5 and AlphaV

**Fig 1:**
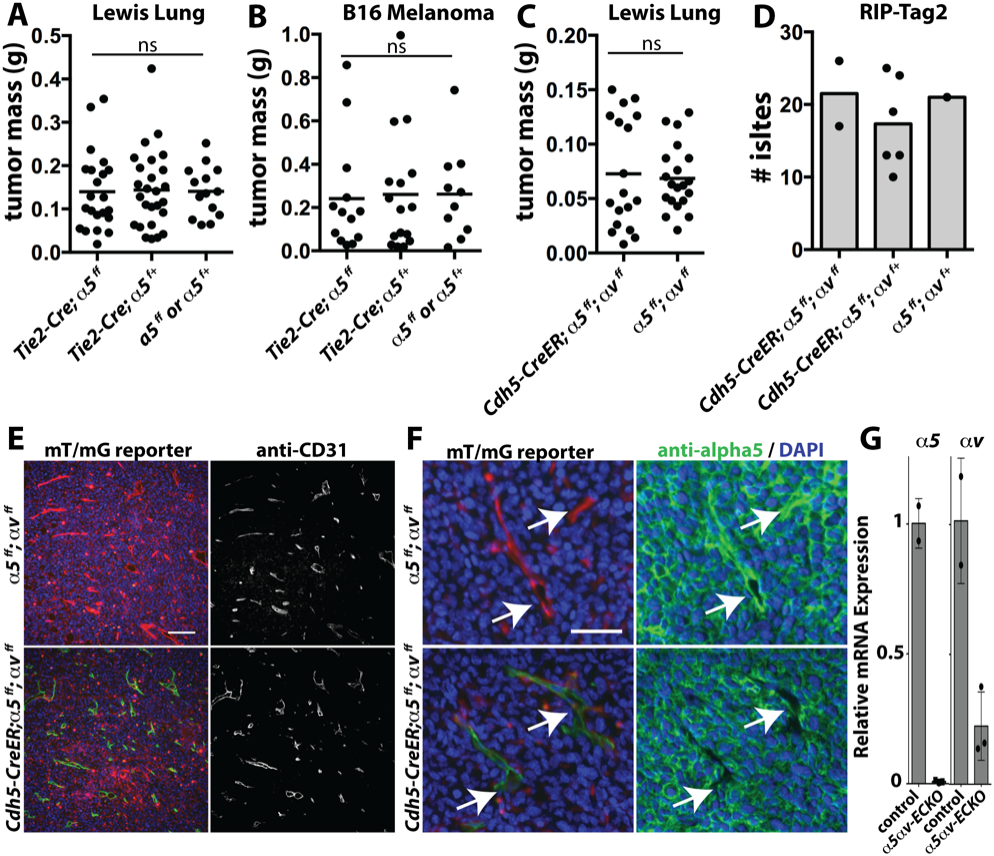
Tumor growth following deletion of integrins Alpha5 and Alphav in the endothelium. (A&B) Tumor mass 12 days after subcutaneous implantation of Lewis Lung (LL2) or 18 days after B16-F10 melanoma cells in the dorsal flank. Each point represents a single tumor, from paired injections into individual mice. (C) Tumor mass 12 days after subcutaneous implantation of Lewis Lung (LL2) cells in mice treated with 3x 1mg tamoxifen 1 week prior to tumor cell implantation. (D) Total number of angiogenic islets harvested from mice with pancreatic neuroendocrine tumours induced by RIP-Tag. (E) Immunofluorescence staining of endothelial marker CD31 or (F) integrin α5 in frozen sections of LL2 tumors from *Cdh5-CreER; mT/mG; α5* ^*f/f*^ *; αv* ^*f/f*^ or *mT/mG; α5* ^*f/f*^ *; αv* ^*f/f*^ mice. (G) Quantitative PCR analysis of RNA isolated from the aortic endothelium of *Cdh5-CreER* (control) or *Cdh5-CreER; α5* ^*f/f*^ *; αv* ^*f/f*^ (*α*5*αv*-ECKO) mice treated one week earlier with 2x 1mg Tamoxifen. Expression of the indicated integrin mRNA, relative to 18s, is normalized to the genetic controls. Points indicate results in individual mice. Scale bars (E) = 100μm, (F) = 50μm.

To determine the requirement for *α5* integrins in tumor angiogenesis, *Tie2-cre* mice were crossed with *α5* ^*f/f*^ mice [11]. We previously found that this results in efficient deletion of endothelial *α5* by embryonic day 10.5 [11]. Despite the absence of endothelial *α5*, we observed no defects in the final tumor mass of either subcutaneous Lewis Lung tumors, or B16 melanoma tumors (Fig. 1A&1B).

Since developmental deletion of *α5* was tolerated, but we had previously found that the combined *Tie2-Cre* mediated deletion of *α5* and *αv* in the endothelium resulted in vascular remodeling defects in the embryo, we hypothesized that *αv* might similarly compensate for the absence of *α5* in tumor angiogenesis [11]. To test this hypothesis, we generated *α5* ^*f/f*^ *; αv* ^*f/f*^ mice, and crossed them with *Cdh5(PAC)-*CreERT2 (hereafter referred to as *Cdh5-CreER*) mice allowing inducible deletion in the endothelium. In these mice, Cre was activated by tamoxifen treatment 1 week prior to tumor inoculation. We found no defect in the growth of the implanted Lewis Lung tumors (Fig. 1C).

Transplant models are highly selected and aggressive, therefore we also examined the requirement for endothelial *α5* and *αv* in the *RIP1-Tag2* (hereafter referred to as RIP-Tag) model of pancreatic cancer [27]. Transformation of the beta cells of the pancreas in this model is induced by insulin-promoter-driven SV40 expression. Cancer growth follows a well-characterized progression from hyperplastic islets at 4–5 weeks of age, to angiogenic islets at about 7 weeks of age, and encapsulated adenomas by 10 weeks of age, and then finally to invasive adenomas by 14 weeks of age [28]. This is a selective process, and of the initial ~400 islets, 50% become proliferative, ~10% become angiogenic and only 1–2% go on to become tumors [29]. The model allows the genetic detection of defects in the angiogenic stage [30] as well as a later growth stage [31].

We treated mice with Tamoxifen at 5–6 weeks of age, and counted the number of angiogenic islets at 10 weeks of age. Here again, we saw no obvious reduction in the number of angiogenic islets, a measure of tumor angiogenesis (Fig. 1D).

We confirmed that *Cdh5-CreER* effectively excises endothelial *α5* and *αv* by several methods. First, we crossed the *mT/mG* Cre reporter line with the *Cdh5-CreER; α5* ^*f/f*^ *; αv* ^*f/f*^ mice. The reporter expresses membrane RFP in all cells from the ROSA locus, and converts to membrane GFP with Cre activation. We induced Cre activity by tamoxifen treatment 1 week prior to Lewis Lung tumor cell inoculation and examined tumor sections for Cre reporter activity, with co-staining for CD31 to confirm the location of endothelial cells. We found that *Cdh5-CreER* induced strong deletion (green) within the endothelium of Lewis Lung tumors (Fig. 1E). Cre activation strongly overlapped with CD31-labeled endothelium, whether or not the floxed *α5* and *αv* alleles were included in the cross (Fig. 1E), suggesting that excised endothelial cells are not selected against in tumor growth. Staining of sections of these tumors for *α5* showed strong *α5* staining in the endothelium of controls, but very little staining in the marked endothelial cells of *Cdh5-CreER; mT/mG; α5* ^*f/f*^ *; αv* ^*f/f*^ mice (Fig. 1F). Furthermore, endothelial expression of *α5* and *αv* mRNA was effectively depleted, as measured in RNA collected from the aortic endothelium of similarly treated mice (Fig. 1G).

Thus, we conclude that *α5* and *αv* are dispensable for tumor angiogenesis. Other integrins (α4β1, α8β1 and α9β1) are able to bind FN, and both α4β1 and α9β1 are expressed on the endothelium, albeit at lower levels. Thus, we cannot exclude the possibility that they are able to compensate for the loss of endothelial *α5* and *αv*.

### Endothelial deletion of Fibronectin does not inhibit tumor growth

**Fig 2:**
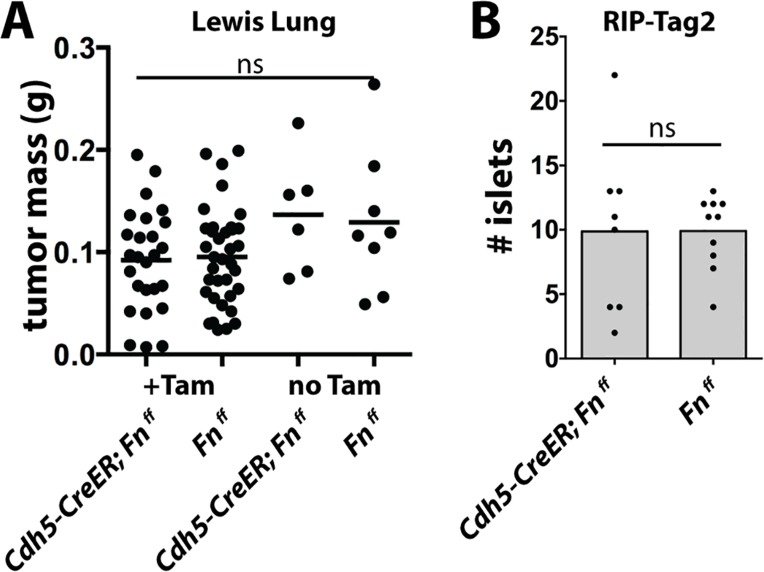
Tumor growth after deletion of endothelial Fibronectin. (A) Tumor mass 12 days after subcutaneous implantation of Lewis Lung (LL2) tumor cells into mice treated with 3x 1mg tamoxifen 1 week prior to tumor cell implantation. (B) Total number of angiogenic islets harvested from RIP-Tag mice at week 10–11.

Given the practical limitations of targeting all of the FN-binding integrins, we turned our attention to FN itself. To determine the requirement for endothelial FN in tumor growth, we used *Cdh5-CreER* to excise *FN* in the endothelium one-week prior to transplant of Lewis Lung tumor cells. We have previously found this results in >90% deletion of endothelial *FN* [32]. The absence of endothelial FN did not significantly affect the final Lewis Lung tumor mass (Fig. 2A).

Similarly, when we treated *RIP-Tag* mice with Tamoxifen at 6 weeks of age, we observed no reduction in the number of angiogenic, red islets in *Cdh5-CreER; FN* ^*f/f*^ mice at 10 weeks of age (Fig. 2B).

Therefore, we conclude that endothelial *FN* is also not required for tumor angiogenesis. This, of course, does not exclude contributions of FN from other sources, since FN is produced by almost all of the cells in the tumor, and is also abundant in plasma.

### Global deletion of Fibronectin delays the formation of angiogenic islets but does not affect final tumor mass

Total *FN* deletion is embryonic lethal, therefore we used *Rosa-CreER* to bypass embryonic lethality and excise *FN* globally in the post-natal mouse. We examined the requirement for *FN* in the *RIP-Tag* model system.

**Fig 3:**
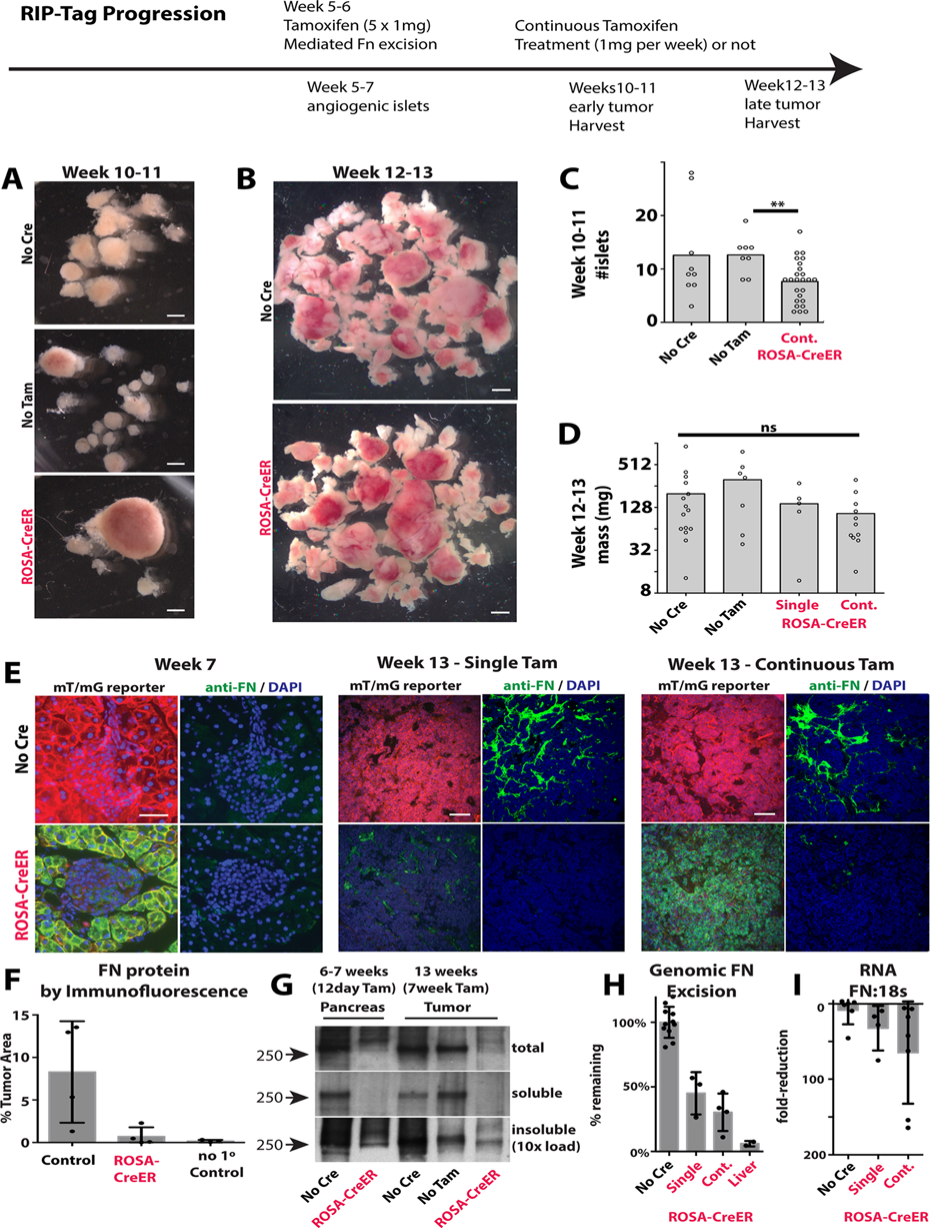
RIP-Tag Tumor growth in the absence of Fibronectin. (A&B) Isolated pancreatic tumors from individual mice. (C) Numbers of angiogenic islets isolated from individual mice. (D) Total mass of tumors isolated from individual mice. (E) Immunofluorescence staining for FN in the pancreas or in tumors in mT/mG reporter mice. (F) Percentage of the total tumor area covered by FN staining in individual mice. Each dot represents the average of three fields from a single mouse. Non-specific control represents the staining from pre-immune serum. (G) Western blot of equal tissue loads (by wet mass) of total pancreas or tumor, and 1% DOC-soluble and insoluble protein. (H) Remaining FN genomic DNA after Cre-mediated deletion, as determined by qPCR relative to an unaffected genomic location. (I) Fold-reduction in FN mRNA expression relative to 18s. Each point represents the results from the largest tumor in each mouse. Scale bars (A&B) = 1mm, (E) 7 week = 50μm, 13 week = 100μm. ** p value <0.01.

We deleted *FN* from the *RIP-Tag* tumors at the angiogenic stage (5–6 weeks) by tamoxifen treatment. To determine the effect of *FN* deletion at the angiogenic stage, we counted the number of angiogenic tumors at 10–11 weeks of age. We found that deletion of *FN* reduced (~30%) the total number of angiogenic islets (Fig. 3A-3C, P<0.004). To determine the effect on final tumor mass, we weighed all of the tumors from the pancreas of each individual mouse at 12–13 weeks. Surprisingly, we saw no significant effect on final tumor mass (Fig. 3D).

To confirm the efficiency of gene deletion, we examined the *mT/mG* Cre reporter in *Rosa-CreER; mT/mG; FN* ^*f/f*^ *; RIP-Tag* mice. We found that excision in the islets and adjacent pancreas was robust 1 week after the first tamoxifen treatment (Fig. 3E). Initially, we had performed analysis with continuous weekly tamoxifen treatment, to prevent outgrowth of cells with intact FN floxed sites. However, analysis of the largest tumors from mice with either a single (5x 1mg, over one week) or continuous (5x 1mg + 1mg weekly thereafter) tamoxifen treatments showed no significant difference in final tumor mass (Fig. 3D), or *mT/mG* Cre-reporter activation at endpoint (Fig. 3E), suggesting that outgrowth of cells with intact *FN* floxed sites does not explain the maintenance of tumor growth.

To confirm the ablation of tumor FN, we examined sections of tumors from *Rosa-CreER; mT/mG; FN* ^*f/f*^ *; RIP-Tag* mice and their Cre-negative or tamoxifen-negative controls at 12–13 weeks. We found the characteristic vascular pattern of FN staining was absent in the *Rosa-CreER; mT/mG; FN* ^*f/f*^ *; RIP-Tag* mice with either single or continuous Tam treatment (Fig. 3E). Quantification of the stained areas revealed a nearly complete ablation of FN in the *Rosa-CreER; mT/mG; FN* ^*f/f*^ *; RIP-Tag* tumors (Fig. 3F).

We asked whether there was any difference in the depletion of DOC-soluble FN versus the DOC-insoluble, or “fibrillar” FN. We found that, consistent with the histological immunofluorescence staining, both total and soluble FN were depleted by 12 days after the first tamoxifen treatment, and that the levels of total and soluble FN remained low to undetectable in most *Rosa-CreER; FN* ^*f/f*^ *; RIP-Tag* tumors up to 13 weeks (N = 3) (Fig. 3G). FN was not entirely removed from the insoluble pool. It is not clear whether the remaining insoluble FN is derived from existing matrix, prior to FN excision, or FN deposited subsequent to FN excision and tumor growth.

We next asked whether FN expression by the tumor cells themselves was also reduced. First, we examined the efficiency in the deletion of *FN* in the tumors by quantitative PCR. We found that Cre activity resulted in a reduction of intact FN alleles by 40–75% with a single period of Tamoxifen treatment and 50–90% with continuous Tamoxifen treatments (Fig. 3H). RIP-Tag tumors were targeted less efficiently than the liver, which yielded a consistent >90% reduction in intact FN alleles with a single Tamoxifen treatment period (Fig. 3H, “liver”). There was no obvious increase in excision frequency with continuous Tamoxifen treatments.

We examined the levels of FN RNA, relative to 18s RNA in the largest tumors of individual mice. We found that FN expression was variable, but that several of the largest tumors isolated from *Rosa-CreER; FN* ^*f/f*^ mice had >150-fold depletion of FN transcript (Fig. 3I).

Thus, we conclude that the genetic deletion of almost all FN in *RIP-Tag* tumors slightly reduces the number of initial angiogenic islets, but does not significantly suppress final tumor mass.

### Angiogenesis and deposition of vascular basement membrane is not suppressed by Fibronectin deletion

*In vitro* studies [9] and *in vivo* developmental studies [1, 2] had suggested FN was essential for angiogenesis, yet apparently normal tumor growth occurred in the near absence of FN. Therefore, we examined tumor angiogenesis closely.

**Fig 4:**
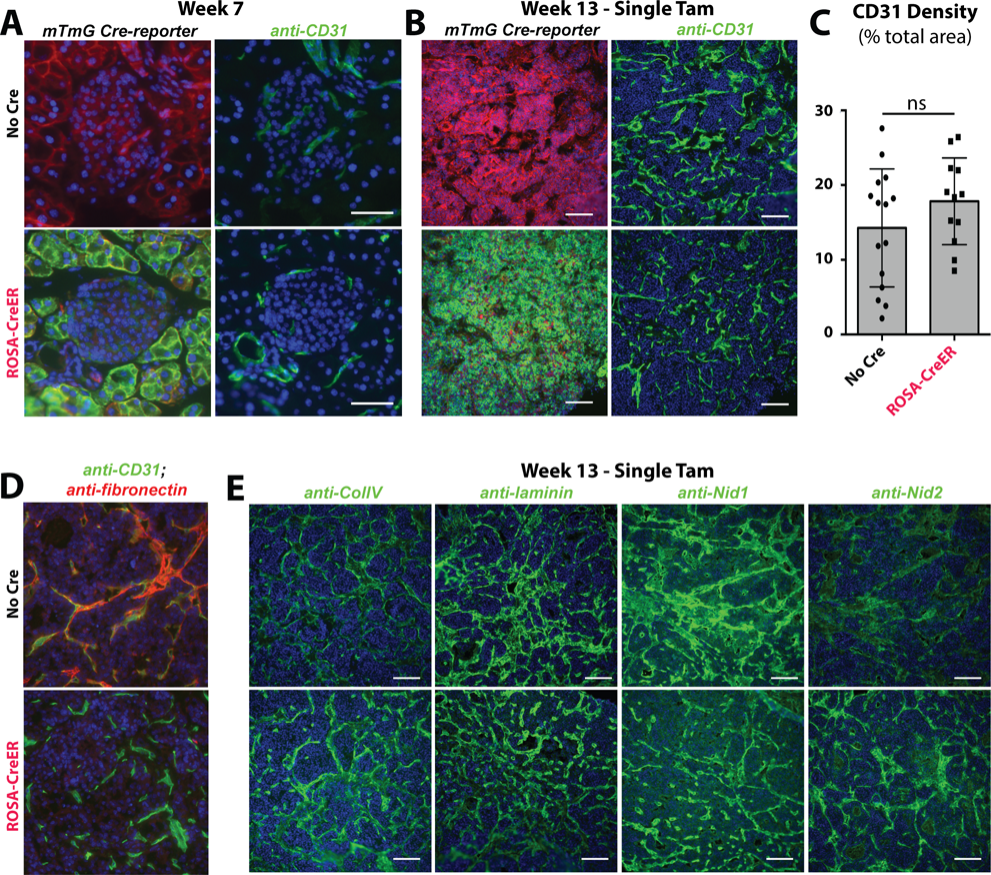
Angiogenesis in RIP-Tag tumors without Fibronectin. (A&B) Immunofluorescence staining of CD31+ vessels in pancreatic islets at 7 weeks and 12–13 weeks after activation of Cre. mT/mG reporter is shown in the same sections, showing the efficiency of Cre activity. Fig. 4A shows the adjacent section to Fig. 3E. (C) Area of the week 12–13 tumors that stained for CD31. The area of each individual field is shown, 3–4 fields per animal (N = 4 per group). (D) Immunofluorescence staining showing co-localization of CD31 and FN in sections from week 12–13 tumors. Each point represents results from a single tumor measurement. (E) Immunofluorescence staining for basement membrane proteins ColIV, laminin, Nid-1 and Nid-2. Scale bars (A) = 50μm, (B&E) = 100μm.

To determine whether the deletion of FN suppressed angiogenesis in the tumors of *Rosa-CreER; FN* ^*ff*^ *; RIP-TAg* mice, we examined CD31 staining by immunofluorescence staining of histological sections of 12–13 week tumors from *Rosa-CreER; FN* ^*f/f*^ mice and *FN* ^*f/f*^ controls. We found no significant reduction in CD31-stained tumor area in the *Rosa-CreER; FN* ^*f/f*^ *; RIP-Tag* mice (Fig. 4A-4C).

FN is typically found adjacent to CD31-stained blood vessels in RIP-Tag tumors, but perivascular FN staining is absent in *Rosa-CreER; FN* ^*f/f*^ *; RIP-Tag* mice (Fig. 4D). Since FN assembly has been shown to be important in basement membrane assembly *in vitro* [10], we tested whether the absence of FN impaired the assembly of the basement membrane in tumors *in vivo*. However, despite the nearly complete absence of FN in the tumor, basement membrane components ColIV, laminin, Nid-1 and Nid-2 appeared to be properly assembled (Fig. 4E).

Thus, we conclude that nearly complete absence of FN in *Rosa-CreER; FN* ^*ff*^ *; RIP-Tag* tumors does not reduce vessel density or appreciably alter the composition of basement membrane components.

### No significant defects in the recruitment of a FN-linked regulator of TGF-beta signaling

*In vitro* studies have shown that FN plays a critical role in the deposition of other ECM proteins, including collagens, fibrillins, fibulins, latent TGFβ-binding proteins (ltbps), and Tenascin-C [8]. However, *in vivo* studies are not as clear. In mice with Mx-Cre-mediated excision of floxed FN in the liver, injury still induces a robust increase in ColI, ColIII, Ltbp-1, Ltbp-3, and Ltbp-4 incorporation into the extracellular matrix [33, 34].

**Fig 5:**
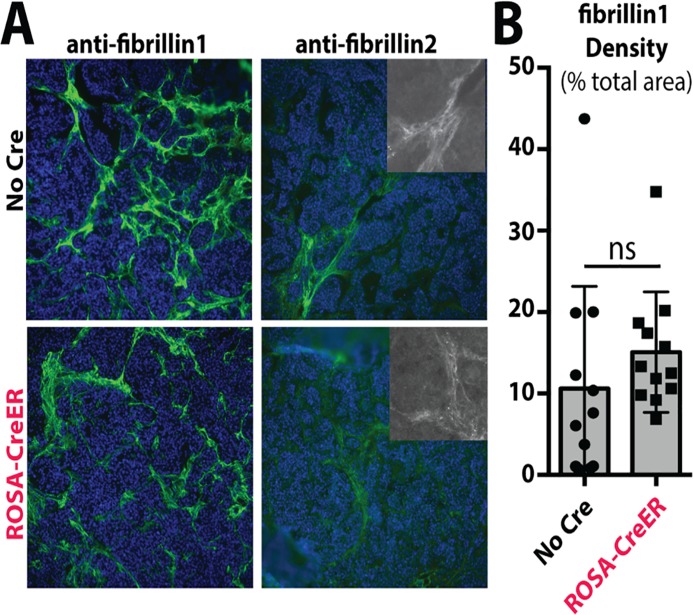
Matrix incorporation of other ECM proteins in the absence of Fibronectin. (A) Immunofluorescence staining of Fibrillin-1 and -2 in 12–13 week RIP-Tag tumors. Insets show fibrils in Fibrillin-2 staining. (B) Quantitation of total tumor area positive for Fibrillin-1 staining. Each point represents results from a single tumor measurement.

We examined the expression of Fibrillin-1, Fibrillin-2, Ltbp-1, Ltbp-3 and Ltbp-4 by immunofluorescence in FN-deleted RIP-Tag tumors. We found abundant Fibrillin-1 expression, and no reduction in the percentage of tumor area with Fibrillin1 staining, relative to control tumors (Fig. 5A&5B). Only minimal levels of Fibrillin-2, Ltbp-1, Ltbp-3 and Ltbp-4 could be detected in tumors of either type, and were not obviously different (Fig. 5A and data not shown). Staining patterns for Fibrillin-1 were similar when detergent was excluded from the staining buffer, suggesting that the fibrillar staining is extracellular matrix and not intracellular deposits (S1 Fig.).

Thus, we conclude that nearly complete deletion of *FN* in *Rosa-CreER; FN* ^*f/f*^ *; RIP-Tag* tumors does not appreciably affect Fibrillin-1 incorporation into the extracellular matrix.

## Discussion

FN and the FN integrin receptors α5β1 and αvβ3/β5 have been thought to be critical for new angiogenesis. Although antibody and small molecule targeting of the adhesion of these receptors to their FN ligand was a promising target for anti-angiogenic therapies, early optimism has been tempered by a series of disappointing clinical trials. However, since lower doses of FN-integrin blocking peptides have been shown to promote, rather than suppress angiogenesis [16], it has not been clear whether the initial pre-clinical success and subsequent clinical failures reflect targeting difficulties or true biological redundancy. Here, using temporally regulated and tissue-specific genetic deletion, we demonstrate that endothelial expression of α5 and αv is dispensable for tumor growth in two transplant models as well as a “spontaneous,” *RIP-Tag*-driven model. Furthermore, we demonstrate that endothelial FN is not required for tumor growth. In fact, nearly complete ablation of FN by *ROSA-CreER*-mediated global deletion does not significantly impede tumor angiogenesis, deposition of basement membrane proteins, or recruitment of matrix-linked proteins Fibrillin-1 and -2 (Figs. 4 & 5). Together, our results demonstrate that the growth of these tumor models is not significantly dependent on FN or its α5β1 and αvβ3/β5 integrin receptors, and suggest that other mechanisms may explain the effects of high-dose RGD inhibitors and integrin-blocking antibodies on angiogenesis observed in pre-clinical studies.

Our data suggest that the antibodies targeting the interaction between FN and its receptors may be doing more than simply blocking this interaction. Blockage of α5β1 binding to FN is a common feature of blocking antibodies—but is not necessarily their only function. Early pre-clinical work showed that antibodies blocking α5β1 binding to FN interfered with adhesion and migration of HUVECs *in vitro*, and potently inhibited FGF-induced angiogenesis in a chick CAM assay [35]. Volociximab, a humanized antibody that interferes with the binding of α5β1 to FN, blocks endothelial cell binding to FN *in vitro* and inhibits angiogenesis in animal models [36, 37]. A similar antibody, designed to bind to murine α5β1 and block its adhesion to FN, blocked angiogenesis in xenografted human tumors, suggesting that effects were mediated by interfering with murine host α5β1, rather than the human α5β1, which was not recognized by the antibody [38]. Yet, our results show that genetic deletion of either endothelial α5 or total FN has little effect on tumor angiogenesis. Differences in models may explain the differential effects. However, a more interesting possibility is that antibodies to α5β1 antagonize tumor angiogenesis not only by a simple block of the interaction between Fn and the α5β1 receptor, but rather by inducing some anti-angiogenic function in the integrin. The genetic deletion of β3 integrin (Itgb3) results in increased angiogenesis and tumor growth [39], while antibody targeting of αvβ3/β5 integrins or mutations of a tyrosine phosphorylation site that leave the β3 receptor intact but disrupt downstream signaling suppresses angiogenesis and tumor growth, suggesting a similar antagonistic effect [40]. While the findings in the RIP-Tag and transplant tumors we describe may not necessarily reflect the requirement for α5β1 and αvβ3/β5 integrins in human tumors, our results suggest that a better understanding of the effects of the blocking antibodies to α5β1 and αvβ3/β5 integrin receptors might be a fruitful path forward towards inhibiting tumor angiogenesis [41].

That robustly vascularized tumors grow in the near absence of FN was surprising, since embryos in which FN has been completely deleted have severe defects in developmental angiogenesis and die by embryonic day 8.5 to 9.5 [1, 2]. Subsequent work showed a requirement for FN in vascular network formation in embryoid bodies [3], and a requirement for the assembly of soluble FN in angiogenesis [9]. Why are the pancreatic tumors, but not the embryo, able to establish vascular networks in the absence of FN? One potential explanation is that FN is not entirely deleted from the tumors, and that the remaining FN is sufficient. Analysis of genomic DNA revealed that deletion ranged from 40–90% in isolated tumors, and reduced DOC-insoluble FN could be detected in Western blot analyses. However, there is effectively no detectable extravascular FN in the tumors, suggesting that if FN is playing a role, it does so as an organizer, acting at low concentrations, rather than simply a “building-block” of the fibrils. Alternatively, the longer time-course (several weeks for tumor angiogenesis, versus several days for embryo angiogenesis) and growing genetic heterogeneity may allow tumors time to compensate for the loss of FN in ways that the developing embryo cannot. This hypothesis is supported by an initial delay (at 10 weeks) in tumor growth in mice deleted for FN that is eventually overcome (Fig. 3). Potential compensating RGD-containing extracellular matrix proteins include the collagens, fibrillins and nidogens, all expressed around RIP-Tag tumor vessels, as we show here. Which, if any, are important in compensating for the loss of FN remains unclear.

*In vitro* studies have suggested a critical function for FN as an organizer of the extracellular matrix, necessary for the integration of other matrix proteins, including ColIV, Laminin, Fibrillin-1 and -2 [8, 10], yet we observed no significant reduction or alteration in the vascular deposition pattern in any of these matrix components in RIP-Tag tumors with nearly complete FN deletion. It is possible that, as with the requirement for FN in angiogenesis, the FN requirement in fibril organization can be achieved at a low concentration, or that its absence only slows, but does not halt matrix assembly. Subtle differences in assembly speed would be more clearly appreciated *in vitro* than in the weeks-long development of RIP-Tag tumors. Another possible explanation is that other matrix proteins could compensate for the function of FN in the deposition of other matrix proteins, as suggested by the initial delay in tumor growth that is overcome at later stages. The ECM produced by growing tumors is diverse, with dynamic changes in the ECM repertoire according to tumor phenotype [42–46]. In liver fibrosis, Col5 has been suggested to compensate for the deletion of liver FN [33]. Whether Col5, or other matrix proteins are able to compensate for the absence of FN in RIP-Tag tumor growth is unclear. Alternatively, compensation may be through changes in ECM-linked signaling pathways, such as Hippo [47]. Further analysis of compensatory measures in this model may reveal new modifiers of vascular matrix assembly.

## Conclusions

Taken together, our results demonstrate that FN and the FN receptors are dispensable for tumor angiogenesis, raising new questions about the mechanisms underlying the anti-angiogenic activities of the blocking antibodies to the FN receptors and underscoring the importance of *in vivo* genetic studies to test the complex regulation of endothelial basement membrane assembly.

## Supporting Information

S1 Fig Staining of Fibronectin and Fibillin-1 in RIP-Tag tumors in the absence of detergent. Immunofluorescence staining of CD31 (green) and Fibronectin or Fibrillin-1 (red) in 12–13 week RIP-Tag tumors in the absence of detergent. Blocking buffer used was 3% Fn-depleted goat serum in PBS. Antibodies and concentrations were as reported in the methods section.
(TIF)
