# Supplementary figures and images for: Tumor Angiogenesis in the Absence of Fibronectin or Its Cognate Integrin Receptors
Source: PLoS One. 2015 Mar 25;10(3):e0120872. doi: 10.1371/journal.pone.0120872 (PMC4373772; doi:10.1371/journal.pone.0120872)

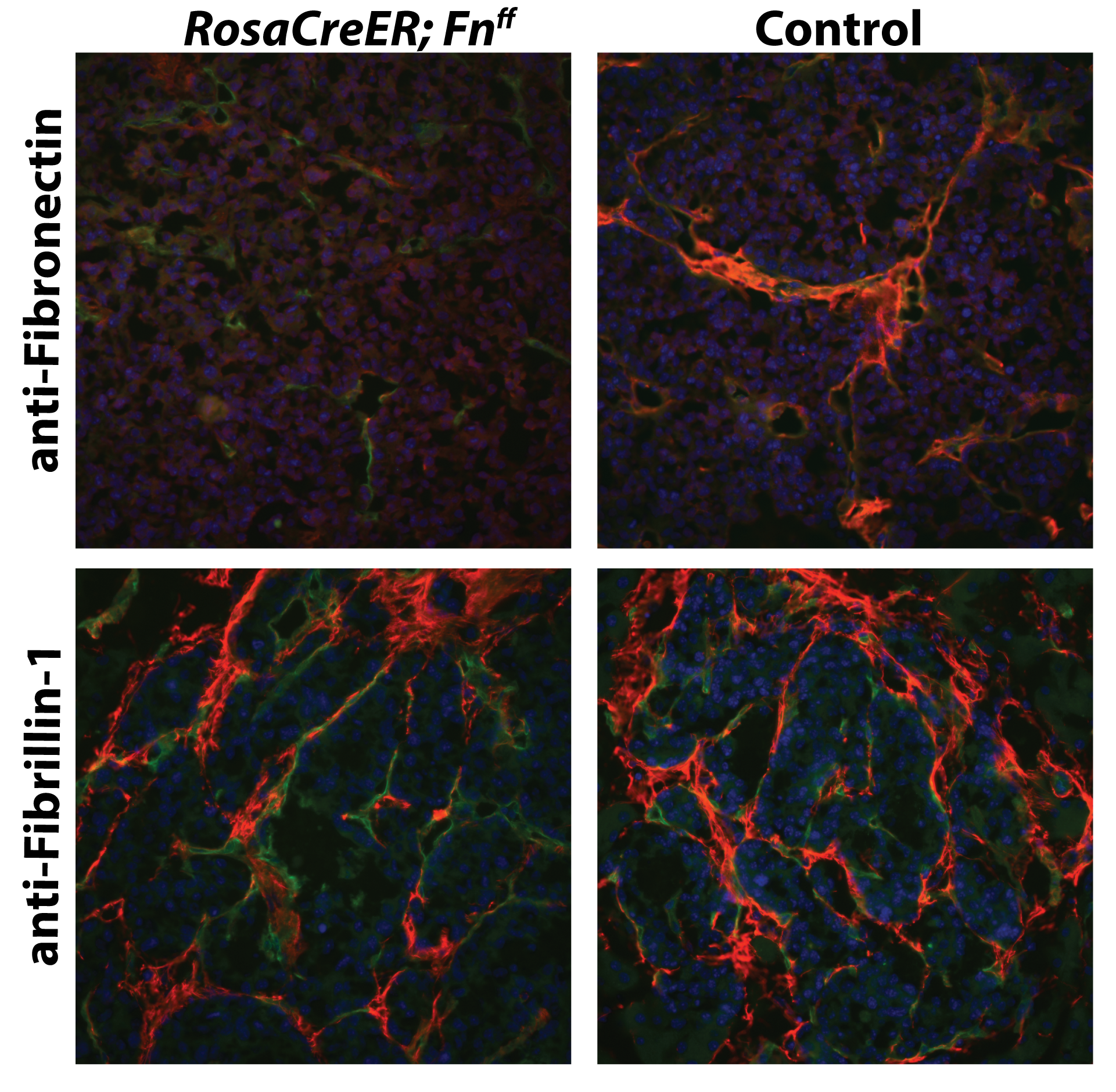

Supplement: S1 Fig — Immunofluorescence staining of CD31 (green) and Fibronectin or Fibrillin-1 (red) in 12–13 week RIP-Tag tumors in the absence of detergent. Blocking buffer used was 3% Fn-depleted goat serum in PBS. Antibodies and concentrations were as reported in the methods section. (TIF) [file pone.0120872.s001.tif]
